# Metabolic Trade-offs in Yeast are Caused by F1F0-ATP synthase

**DOI:** 10.1038/srep22264

**Published:** 2016-03-01

**Authors:** Avlant Nilsson, Jens Nielsen

**Affiliations:** 1 Chalmers University of Technology, Department of Biology and Biological Engineering, Gothenburg, SE41296, Sweden; 2 Technical University of Denmark, Novo Nordisk Foundation Center for Biosustainability, Hørsholm, DK2970, Denmark

## Abstract

Intermediary metabolism provides living cells with free energy and precursor metabolites required for synthesizing proteins, lipids, RNA and other cellular constituents, and it is highly conserved among living species. Only a fraction of cellular protein can, however, be allocated to enzymes of intermediary metabolism and consequently metabolic trade-offs may take place. One such trade-off, aerobic fermentation, occurs in both yeast (the Crabtree effect) and cancer cells (the Warburg effect) and has been a scientific challenge for decades. Here we show, using flux balance analysis combined with *in vitro* measured enzyme specific activities, that fermentation is more catalytically efficient than respiration, i.e. it produces more ATP per protein mass. And that the switch to fermentation at high growth rates therefore is a consequence of a high ATP production rate, provided by a limited pool of enzymes. The catalytic efficiency is also higher for cells grown on glucose compared to galactose and ethanol, which may explain the observed differences in their growth rates. The enzyme F1F0-ATP synthase (Complex V) was found to have flux control over respiration in the model, and since it is evolutionary conserved, we expect the trade-off to occur in organisms from all kingdoms of life.

To generate energy, yeast can use both the, oxygen consuming, respiratory pathways and the, ethanol producing, fermentative pathways. The respiratory pathway generates 10 times more ATP per glucose molecule, and has a fivefold higher biomass yield^1^. It is therefore somewhat counterintuitive that yeast makes use of the fermentative pathway even in the presence of oxygen, i.e. the Crabtree effect^2^, when it grows fast. Similar examples of aerobic low yield metabolism occur in other microbes, e.g. *E. coli*^3^, and in human^4^, e.g. lactate production in cancer, muscle and red blood cells. The underlying biological motivation for this is still under debate, and it is not predicted by standard metabolic models^5^. But it has been hypothesized that fermentation has a higher catalytic efficiency than respiration, i.e. that more energy is produced per mass of enzyme^6^.

Many microorganisms and mammalian cells use glucose as their preferred carbon source, but can still utilize a range of alternative carbon sources. Catabolism of galactose through the Leloir pathway only involves a few additional enzymatic steps, but the growth rate is much lower^1^. Over-expression of the enzymes of the Leloir surprisingly decreased the growth rate even further^7^. This has been attributed to the physiological burden on the cell from the transcription and translation of additional enzymes^8^. Yeast cells grown on glucose in batch culture^9^ first deplete the glucose whilst producing ethanol. They then undergo a diauxic shift, where their protein composition is remodeled, and continue to growth on ethanol, at a lower rate.

To understand these observations, we turned to the hypothesis that cells are constrained by the allocation of protein mass. This hypothesis has gained recent attention through the description of the cell as a self-replicating system^6^,^10^, where different cellular processes, e.g. metabolism, biosynthesis, ribosomes, membrane transport, compete for a finite protein pool in order to maximize growth. The proteome of *E. coli*, has been found to cluster in to such biologically distinct sectors, with positive or negative relations to the growth rate^11^. The protein pool becomes limiting, since each enzyme has a characteristic mass 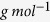 and effective rate 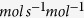, with a resulting mass efficiency, i.e. the enzymes specific activity 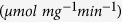. This has also been studied from the perspective of molecular crowding^12^, where the total amount of enzyme is limited by the solvent capacity of the cytosol. But since the solvent capacity is shown to be near invariant^13^, this is similar to a limited protein pool.

An extension of flux balance analysis^12^,^14^ has been developed to handle this catalytic dimension of metabolism. These models use the specific activities of the enzymes to either estimate their volume, constrained by a limited solvent capacity^12^, or more directly by estimating their share of mass in the total protein pool^14^. The method has been successful in predicting the maximum growth rate of *E. coli* on different carbon sources^14^. And it also has the potential to explain aerobic low yield metabolism, since genome scale models with randomly sampled data predict this in their best fit, in both *E. coli*^12^ and *S. cerevisiae*^3^. This was also the predicted outcome in a genome scale model of human, using *in vitro* enzyme activity for 15% of the reactions and the median for the remaining^15^. In this study we reduce the scope to intermediary metabolism of *S. cerevisiae* to avoid the use of simulated data and ensure complete *in vitro* enzyme data coverage.

In *S. cerevisiae* the size of the protein pool depends on the growth rate, and varies between 40–60% of the dry weight^2^,^16^. The remaining mass is allocated to RNA 6–12%, glycogen 0–8% and the cell wall of polysaccharides 10–25%^16^,^17^. A large share of the protein pool is taken by enzymes involved in protein synthesis, e.g. the ribosomes, and they are responsible for most of the changes in protein mass (Supplementary Figure S1). Measurements of the growth rate dependent changes in biomass composition show that *S. cerevisiae* can increase its protein content (g per g cell dry weight) by decreasing the glycogen reserves^16^. It can also increase the protein content by increasing the cell size, since this increases the protein rich cytosol at the expense of the cell wall, by shifting the ratio of surface to volume (Supplementary Figure S2). Increasing cell size, however, has a trade-off, as it may reduce the substrate uptake, by the decreased the surface to mass ratio (Supplementary Figure S2). As the growth rate increases, so does the requirement of energy and precursor metabolites generated in the intermediary metabolism, but due to the increasing requirement for ribosomes this has to be provided using a constant or even shrinking pool of protein mass allocated to the enzymes involved in intermediary metabolism (Supplementary Figure S1), and this becomes a challenging optimization problem.

## Results

### The Model Predicts the Crabtree Effect

**Figure 1:**
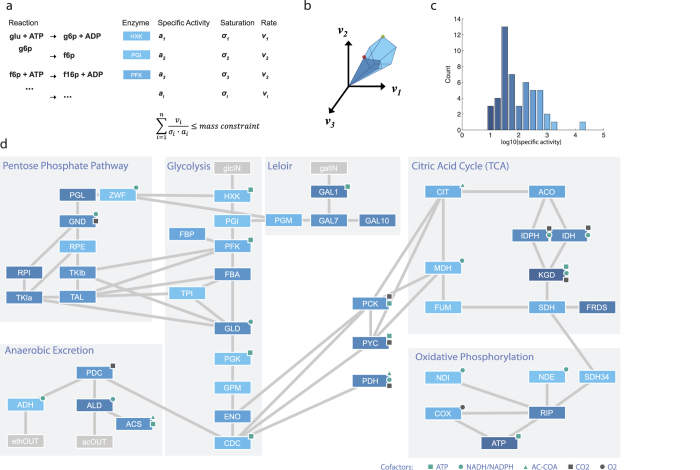
The model of intermediary metabolism. (**a**), Each enzyme, *i*, may carry a flux, *v*_*i*_, 
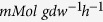
 and operates with an activity, 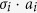, where *a* is the maximum specific activity of the enzyme 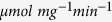 and 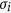 is a saturation factor. The sum of mass is constrained. (**b**) The constraint shrinks the subspace of feasible fluxes, which may result in a different optimal solution. (**c**) Histogram of log10 transformed specific activity values from the model 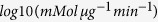. (**d**) Enzymes are connected if they share a metabolite, and are colored according to their specific activity (**c**).

To study the effects of protein efficiency, we implemented a stoichiometric model of the intermediary metabolism (Fig. 1), including a biomass equation. We then collected the *in vitro* specific activities 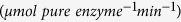, for all of the enzymes in the model (Supplementary Table S1). This allowed us to estimate the mass of each enzyme required to support metabolic flux. We then used proteomics data from literature^18^ to calculate the fraction of protein mass in intermediary metabolism (200 mg/g protein). Together with a typical protein content of *S. cerevisiae* of approximately 50%^2^, the mass of protein covered by the model was estimated to 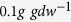, and we constrained the model to this value.

**Figure 2:**
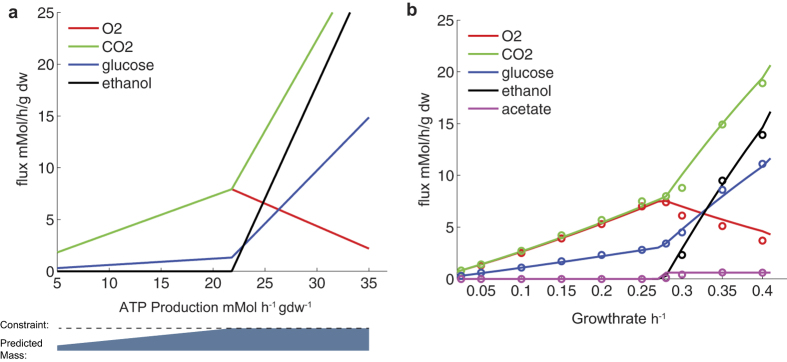
The exchange fluxes of metabolites depend on the ATP production rate (a) and growth rate (b). Simulations were run with the objective to minimize substrate utilization at increasing production rates. At low rates the protein mass is not limiting, as illustrated by the predicted mass bar. At a critical rate the fluxes become large enough for the constraint to come in to effect, and a switch in metabolic strategy occurs. Fermentation gradually replaces the less mass efficient oxidative phosphorylation, with increasing rates, as this is the most substrate efficient strategy consistent with the mass constraint. Experimental data^2^ 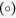 is overlaid the model predictions (−).

We observed that fermentation requires less protein per produced ATP than oxidative phosphorylation (Supplementary Table S2). Together with the mass constraint, this leads to a critical ATP production rate, after which fermentation gradually replaces respiration (Fig. 2a). Since biomass production depends on ATP, this translates into a critical growth rate at which ethanol production is onset, the key characteristics of the Crabtree effect. This is consistent with the observation that the critical growth rate is lowered when the ATP expenditure is increased^19^. It is also consistent with the inverse correlation between the critical growth rate and the nitrogen content of the medium^20^, since nitrogen starvation should reward protein efficiency. An increased protein efficiency should also decrease the amount of ribosomes required for growth. This may explain that the Crabtree effect is promoted by phosphor limitation^21^, since ribosomal RNA is the main sink for phosphor at high growth rates.

We used the model to simulate yeast metabolism in a chemostat operated at different dilution rates (equal to the specific growth rate), and found good agreement with experimental values (Fig. 2b). To better fit the experimental data, the ATP expenditure was adjusted to follow the experimentally determined levels (See Supplementary Figure S3 for the steps of the fitting process and the growth rate dependence of the ATP expenditure). The observed variations in ATP expenditure may be explained by shifts in the biomass composition, e.g. in the experimental setting^2^ the protein level was observed to increase from 40% to 53% below the critical growth rate, and then decrease again to 46%.

Chemostat experiments have shown that yeast produces acetate at high growth rates^2^. The model correctly predicted the critical growth rate at which this occurs, and that the rate of acetate production stabilizes. The flux was initially over predicted, 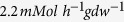 compared to 0.6, which is in better agreement with experiments in batch culture, where rates up to 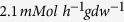 are observed^22^. To avoid an incorrect carbon sink we put an upper bound on the acetate production at the experimentally observed level. We then used the model to investigate the underlying mechanism behind the acetate production, and found that it was related to anabolic reduction power, NADPH. As fermentation replaces respiration, NADPH production by the pentose phosphate pathway (PPP) is replaced by the acetate producing aldehyde dehydrogenase (ALD6), because the cell needs a sink for carbon from glycolysis. This shift is confirmed by C13 labeling experiments^23^.

### F1F0-ATP Synthase Causes the Crabtree Effect

**Figure 3:**
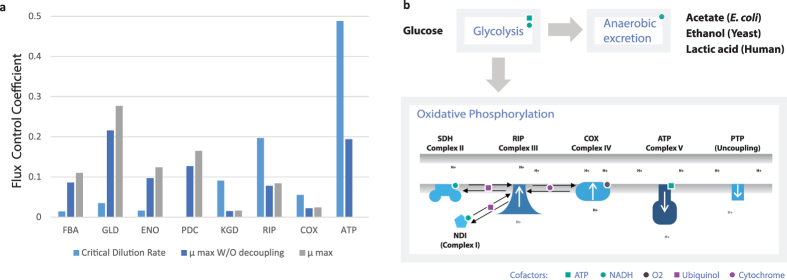
(**a**) Flux control coefficients (FCC) quantify the relation between enzyme activity and the predicted growth. The critical growth rate is dominated by enzymes from oxidative phosphorylation, and the maximum growth rate by enzymes from glycolysis. Figure shows enzymes for which 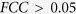, a full list is also available (Supplementary Table S3). (**b**) A branch point between oxidative phosphorylation and anaerobic excretion is present in several organisms, with organism specific fermentation products. The rate of ATP formation by F1F0-ATP synthase (complex V) is given by the proton motive force. This force can be uncoupled using the permeability transition pore (PTP) which allows Complex I–IV to use oxygen as an electron acceptor unconstrained by the low activity of Complex V.

To gain mechanistic insight into the Crabtree effect we investigated how the critical and maximum growth rates depend on the specific activity parameter of each enzyme (Fig. 3a). The enzymes with high flux, but low specific activity, exerted most control. The critical growth rate was mainly controlled by enzymes from oxidative phosphorylation, most noteworthy F1F0-ATP synthase contributed with almost half of the flux control. Several enzymes shared control over the maximum growth rate, with the common denominator that they could not be bypassed using the fermentative growth strategy.

Because of the high flux control of F1F0-ATP synthase, we expect that the cell regulates the amount of this enzyme tightly. Above the critical rate this is necessary, since it is the predicted decrease in F1F0-ATP synthase that allows more protein mass to be allocated to the glycolytic and fermentative enzymes. Proteomics of the mitochondria show that the relative abundance of proteins within the mitochondria is independent of respiratory or fermentative conditions^24^, F1F0-ATP should therefore be proportional to the mitochondrial content. Microscopy analysis reveals that mitochondria increases three fold in volume, when respiring on glycerol compared to fermenting on glucose^25^. Experimental studies of respiratory capacity show that yeast adjusts the amount of mitochondria to match the growth rate dependent ATP synthesis rate^26^. Mitochondria has also been shown to increases as a response to increased ATP expenditure due to the addition of Benzoic acid, and can cover up to 26% of the cell volume^27^.

We investigated the robustness of the Crabtree prediction by withholding each enzyme from the mass constraint in turn. F1F0-ATP synthase was the only enzyme that single-handedly changed the result. To verify the parameter value, we used the fact that F1F0-ATP synthase is evolutionary conserved^28^, and searched the literature for F1F0-ATP synthase activity measurements from other organisms (Supplementary Table S4). The electrochemical gradient, i.e. the proton motive force (PMF), is the most influential factor in determining the activity of the enzyme. At cellular levels of PMF, 180–220 mV^29^, the specific activity from 10 different experiments was found to be 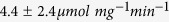, which is comparable to 5.6 used in the model. We also noticed that the turnover number of F1F0-ATP synthase in yeast^30^, 120 *s*^−1^, was almost twice as high as the median turn over number in the model, 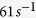, but that its specific activity was one of the lowest, due to its molecular mass being more than 10 times higher than the median, 640 kDa compared 55 kDa (Supplementary Table S1).

The evolutionary preservation of F1F0-ATP synthase likely explains the universality of low yield metabolism. A challenge to this are the Crabtree negative yeasts, e.g. *K. marxianus*^31^ that only produces low amounts of ethanol even at as high growth rates as 0.5 *h*^−1^. This might be explained by their high protein content^31^, up to 72%, compared with only about 46% in *S. cerevisiae*^2^, since the Crabtree effect is only predicted to occur if protein is limiting. The protein content of the two organisms are similar up to the critical growth rate after which they diverge (Supplementary Figure S1). Although the respiring *K. marxianus* grows faster than *S. cerevisiae,* it still grows slower than the fermenting *S. kluyveri*, 0.63 *h*^−1 ^31.

### Implications For Oxidative Phosphorylation

A major difference between oxidative phosphorylation in *S. cerevisiae* and most other organisms is that the large hydrogen pumping protein complex, Complex I, 1000 kDa^32^, is replaced by a single enzyme NDI, 56 kDa^33^. NDI does not contribute to the proton gradient, and has a 40 times higher specific activity compared to its human counterpart^32^,^34^. Complex I’s contribution to a high ATP yield on glucose makes it important for organisms surviving conditions of glucose starvation, e.g. many filamentous fungi and humans. But it likely limits the ATP production rate for fast growing organisms, since it is better to allocate the mass to F1F0-ATP synthase according to our analysis of flux control. A similar phenomenon occurs in *E. coli* at high growth rates, where the proton pumping Complex I is replaced by a non pumping variant^35^.

When producing ATP under fermentative conditions, the NADH formed by glycolysis is balanced with the consumption from alcohol dehydrogenase (ADH). However, when the cell is growing, some of the carbon is diverted to biomass and there is a net production of NADH^36^. This can be resolved by producing glycerol or through oxidative phosphorylation. Since F1F0-ATP synthase limits the flux through oxidative phosphorylation, we investigated the effect of adding an uncoupling reaction to the model, allowing it to bypass F1F0-ATP synthase and release the proton gradient (Fig. 3b). Such uncoupling has been observed in isolated yeast mitochondria^37^ and takes place in yeast through the permeability transition pore (PTP), which has recently been identified as an uncoupled state of F1F0-ATP synthase^38^. The addition of the uncoupling reaction increased the predicted maximum growth rate by 4%, the ethanol production rate by 30%, and decreased the biomass yield by 15%. This type of low yield growth is observed in batch cultures, and is likely triggered by the presence of high glucose concentrations. Together with the formation of additional fermentation products and increased ATP expenditure it may explain the 80% higher biomass yield observed in chemostats at the same specific growth rate (Supplementary Table S5). This is consistent with the observation that the proton gradient, 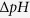, between cytosol and mitochondria is 0.3 in batch cultures and 0.5 when respiring in glucose limited chemostats^39^.

### Prediction of Enzyme Abundances

**Figure 4:**
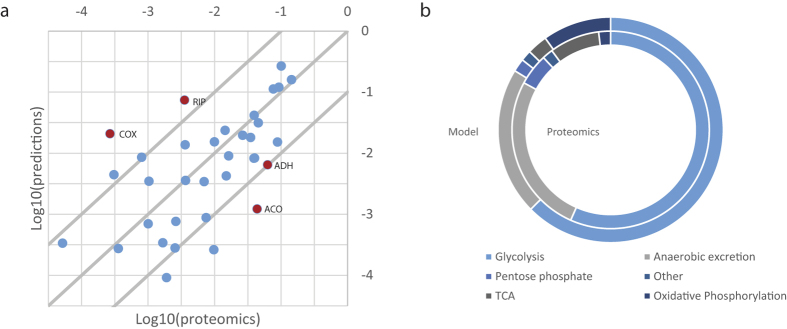
Comparison of the protein mass ratios predicted by the model and from proteomics abundances^18^,^59^ multiplied with tabulated masses^33^. (**a**) A Log10-Log10 plot of the data shows that most predictions lie within an order of magnitude of the proteomics measurements. Miss matches are highlighted in the cases where either the predicted or proteomics values are of significant magnitude, 1% (−2 on the log scale) of the mass or higher. For 10 reactions no flux was predicted and therefore no protein abundance, this corresponded to 6% of the proteomics data. Non log transformed data can be found in Supplementary Table S6. (**b**) the distribution of mass between pathways.

The model assumes that all enzymes operate at 0.5 of their maximum specific activity. This assumption is based on the observation that most glycolytic enzymes are half saturated in *E. coli*^40^, and on the trade-off between low metabolite concentrations and high enzyme saturation^41^. This is expected to be incorrect for almost all enzymes, since each saturation factor will depend on allosteric regulation, phosphorylation state, internal metabolite concentrations and in some cases on strong backwards fluxes due to thermodynamics. We confirmed this suspicion by comparing the predicted protein mass to proteomics data from literature (Fig. 4). For the individual enzymes the predictions were not very accurate (Fig. 4a), although most predictions lie within an order of magnitude of the protein abundance measurements. On the level of pathways these errors however appear to cancel out (Fig. 4b).

One of the more striking under predictions occurred for alcohol dehydrogenase (ADH), this is likely due to product inhibition in the batch culture (Fig. 4a). Aconitate hydratase (ACO) was also strongly unpredicted, possibly because the backwards reaction is thermodynamically favored^42^. Two enzyme complexes from OXPHOS (RIP and COX) were in contrast over predicted by the model. This could be related to detection problems for membrane bound complexes, but could also be an additional indication that the electron transport chain is uncoupled under batch conditions.

### The Model Predicts Substrate Dependent Maximum Growth Rates

**Figure 5:**
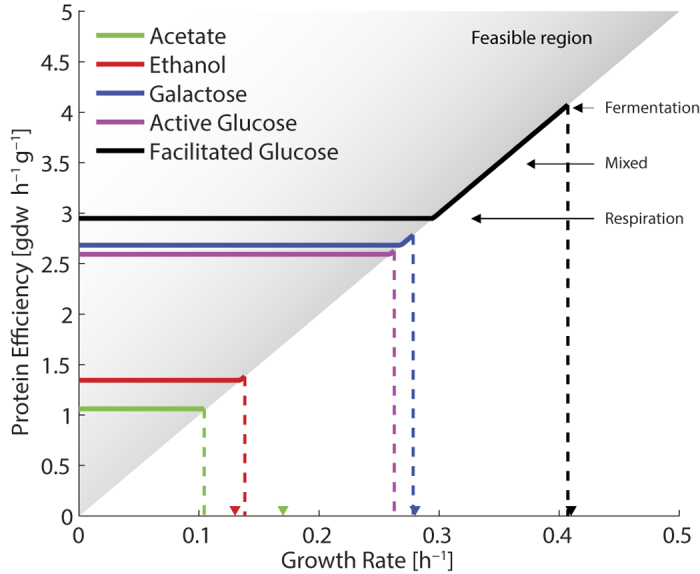
The simulated protein efficiency for growth of biomass on different carbon sources, and the predicted maximum growth rates. Increasing growth rates require more efficient protein, assuming a fixed enzyme pool 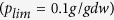, resulting in the feasible region 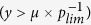. The enzymes involved in the utilization of ethanol and acetate have low calculated efficiency, and therefore the maximum growth rate is predicted to be low. For glucose and galactose the gradual shift towards fermentation at high growth rates increases the protein efficiency and thereby the predicted maximum growth rate. Predicted rates are compared with experimentally observed growth rates^1^ (▾), acetate 0.11 (0.17), ethanol 0.14 (0.13), galactose 0.28 (0.28) and glucose 0.41 (0.41) *h*^−1^.

Distinct metabolic pathways are utilized for growth on different substrates, e.g. glucose, galactose, acetate and ethanol. Using the model we predicted the maximum specific growth rates (Fig. 5) and found them to be in good agreement with experimentally determined rates^1^. The Crabtree effect is predicted to occur for growth on both glucose and galactose. The lower efficiency and predicted growth rate for galactose is due to the burden of the additional and relatively inefficient enzymes of the Leloir pathway (Fig. 1d). These enzymes are especially burdensome during fermentation where the glycolytic flux is increased. The shift to fermentation is therefore predicted to occur over a shorter range of growth rates. We also noticed that the model predicts the production of acetate when growing on ethanol at high rates, which is normally observed towards the end of experiments in batch culture^9^.

The model correctly predicts a significantly lower growth on ethanol and acetate compared with glucose. And the lower efficiency for these substrates is caused by a higher degree of respiration and thereby ATP generated through F1F0-ATP synthase, according to our analysis of flux control (Supplementary Table S3). The model however failed to predict their internal order. This might be a consequence of inconsistent substrate concentrations in the experiment^1^, but may also be related to the model underestimating the mass allocated to ADH (Fig. 4). The total amount of mass that can be allocated to intermediary metabolism might also be underestimated at these low growth rates, since the ribosome content is growth rate dependent (Supplementary Figure S1).

We investigated the effect of active glucose transport by adding a transport reaction for glucose requiring 1 mol ATP per mol glucose. This had only a a minor effect under respiratory conditions, the ATP produced per mass decreased by 6%. But for fermentation it reduced the efficiency by 50%, since the net production of ATP decreased to 1 ATP per glucose, which made it a less viable growth strategy than respiration. This may explain the dominance of glucose transporters using facilitated diffusion in yeast^43^.

## Discussion

Metabolic modeling of metabolic trade-offs has a long history. It was early suggested that a limitation in respiratory capacity could explain the shift ethanol production, when the uptake of glucose increased^44^. Using the cybernetics approach, the overflow metabolism in *E. coli* was linked to a limitation in respiration or the production of NADH by the TCA cycle^45^. These limitations were later given a biophysical motivation, by macromolecular crowding^12^ or enzyme mass allocation^14^. The experimentally determined specific activity values, used in this study, point towards a limitation in respiration, as fermentation appears to be a more protein efficient strategy. This result could be expected based on chemostat studies^2^, where the ratio of ATP production per total cell protein increases by 40% between respiration at growth rate 0.25 *h*^−1^, and fermentation at growth rate 0.4 *h*^−1^, due to the simultaneous increase in ATP production (Supplementary Figure S3) and decrees in protein content (Supplementary Figure S1). From flux analysis perspective this is however not the expected outcome, since the sum of fluxes (often used as a proxy for enzyme usage^46^) in contrast is 70% higher for ATP production by fermentation, compared to respiration (Supplementary Table S2). It is only by weighting the sum of fluxes with the specific activities that fermentation becomes more efficient, illustrating the value of using experimentally determined parameters. By making enzyme allocation a part of the flux cone (Fig. 1b), we get a rational for the mixture between respiration and fermentation observed at intermediate growth rates 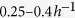 (Fig. 2b). These are otherwise considered elemental flux modes, and optimality would suggest using one or the other^47^.

The perspective of optimal enzyme allocation is challenged by experiments showing that cells keep substantial amounts of enzymes available also in conditions that do not strictly require them^48^ and that fluxes through glycolytic enzymes appear to be regulated on a post transcriptional level^49^. This may be understood as an adaptive strategy, where the cell is prepared to quickly respond to changes in environmental conditions^48^. And after prolonged selection in a glucose limited chemostat, there is indeed a significant decrease in glycolytic activity^50^. An additional glycolytic capacity is compatible with the present study, since less efficient protein allocations are within the feasible region at sub maximum growth rates (Fig. 5). A study of the growth rate dependence of glycolytic enzymes in the laboratory strain CEN.PK^51^ show that they in general decrease as the critical growth rate is approached, and then increase again towards the maximum rate. For the industrial strain, investigated in the same study, there was no such trend and the fermentative capacity was consistently higher. The glycolytic capacity might also be influenced by growth rate dependent differences in intracellular metabolite concentrations. Systematically lower concentrations would decrease the saturation of each enzyme, and call for increased enzyme abundances. This illustrates that the saturation factor, *σ*, assumed to be 0.5 for all enzymes, is a simplification. A more refined model would have a unique, condition dependent, saturation factor for each enzyme, as has recently been suggested^47^. Experimental fitting of such saturation parameters would however be a challenge and the increase in number of parameters might result in over fitting.

The predicted maximum growth rate and critical growth rate depend linearly on the protein constraint, 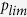 and saturation factor *σ* since FBA is a linear equation system. They are also proportional to the ATP production, since it is the main cause of flux and thereby the predicted enzyme abundances. In the current formulation where *σ* is assumed to be the same for all enzymes, 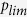 and *σ* can be replaced by a single variable. Although this is a free variable from mathematical perspective, the usage of proteomics data and reasoning about general enzyme saturation levels confines it to a limited range. For *σ* the upper bound is 1 corresponding to the scenario where all enzymes are at constant maximum saturation. There is no corresponding lower bound, but from an evolutionary point of view it would be wasteful for it to be much lower than 0.5, since kinetic parameters can be adjusted on evolutionary timescales, within their physical limitations, to fit the needs of the organism. As has been shown in a cross-species analysis^52^. The 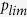 depends both on the relative protein abundances between different subsystems, and on the total protein content of the cell. The total content is relatively similar in the cases where 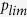 is in effect, 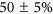. The proteomics data used to estimate the fraction, 20%, that goes in to intermediary metabolism is generated for cells grown on glucose in batch condition, and might not necessarily be valid for other conditions. These conditions are however relatively similar to chemostats at high dilution rates, and the functions of the remaining subsystems should be necessary also under other conditions. Protein synthesis is the most abundant subsystem 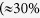 of the mass) and its abundance increases with growth rate (Supplementary Figure S1), as it has been shown to do in *E. coli*^53^. This means that 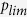 might be increasingly underestimated at lower growth rates. This effect should however be limited, since a substantial share of the protein pool is allocated to protein synthesis also at low growth rates (Supplementary Figure S1). Although protein synthesis takes a large share of the protein pool in *S. cerevisiae*, this share, and there by its relative importance, is much lower than in *E. coli*^53^, due to the significantly lower maximum specific growth rate 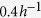 compared to 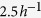.

The experimentally determined specific activity values are an additional source of uncertainty. The error is normally reported to be small within the experiment, but searching the enzyme database BRENDA^54^, it is not uncommon to find that experimental values differ between labs by a factor of 2 or more. To test the effect of different saturation levels and experimental noise we ran the simulation 5 000 times with randomly perturbed specific activity values. The sensitivity analysis shows that the predictions of the Crabtree effect and maximum growth rate are robust (Supplementary Figure S4). This is a consequence of the specific activity values having a logarithmic distribution (Fig. 1c), where large perturbations are required to disrupt the internal order.

We here present a stoichiometric model of intermediary metabolism, augmented with *in vitro* enzyme activity data, that quantitatively predicts the Crabtree effect as well as the maximum growth rate on different carbon sources. The taken approach has obvious advantages over standard FBA, that predicts neither, and over kinetic models, due to its comparatively low complexity. Using the model we identified F1F0-ATP synthase as a key flux controlling enzyme for respiratory metabolism in yeast, which provides new insight into the evolutionary advantage of the Crabtree effect. Given the strong predictive power of the model, together with its simplicity we project that this approach has great potential for studying also other parts of metabolism, e.g. amino acid biosynthesis that involves reactions in both cytoplasm and mitochondria. But also for the field of metabolic engineering where it may be used to evaluate the impact of introducing a synthetic pathway that has to compete for the total protein pool.

## Methods

### Model formulation

A stoichiometric model is, briefly, a sparse matrix where the rows correspond to metabolites and the columns to reactions. In this formulation biomass is defined as the pool of metabolites that are required for growth and these are gathered using an artificial biomass reaction. The model is transformed into a linear optimization problem using the principles of flux balance analysis (FBA)^55^. FBA investigates flux distributions that fulfill the constraint of homeostasis, i.e. steady state metabolite concentrations. The model assumes that the cell will chose the flux distribution that maximizes growth for a given substrate uptake rate^46^, or equivalently minimizes substrate uptake for a given growth rate^55^.

A stoichiometric model of intermediary metabolism, iAN50, was implemented based on the genome scale iFF708 model^55^ and a model of yeast intermediary metabolism from the RAVEN toolbox^56^. The model included glycolysis, the pentose phosphate pathway, anaerobic excretion, citric acid cycle (TCA), oxidative phosphorylation, and uptake pathways for galactose, ethanol and acetate. The galactose uptake reactions from the iFF708 model were corrected using the KEGG database^57^. The drain of metabolites from intermediary metabolism to the biomass equation were estimated using the iFF708 model. A simulation minimizing glucose uptake was run in the iFF708 model. The directionality of all the fluxes was fixed, and the simulation was run again allowing free uptake of all the metabolites found in iAN50 but disallowing glucose uptake. These free uptake fluxes were minimized and taken as the drain of metabolites on iAN50 for the biomass equation. Setting this as the biomass equation ensured that the fluxes were the same for the corresponding reactions in iAN50 and iFF708 model. In addition the equation was made modular so that biomass constituents, e.g. protein, RNA and DNA could be optimized one by one.

In extension to standard FBA, the total enzyme mass was estimated and constrained to the biologically observed level, using an approach equivalent to one taken before^12^,^14^. Briefly, for each reaction the mass of the enzymes required was estimated by dividing the predicted flux 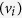 with the maximum *in vitro* activity 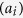 and a saturation factor 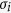. In this simulation 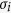 was fixed to 0.5 for all reactions. To ensure that all 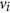 were positive, each reversible reaction was split in to two irreversible reactions in each direction. The *in vitro* activities where collected from literature, and all though most of the studies were found using the enzyme database BRENDA^54^, the individual publications were investigated and the values taken directly from the source (Supplementary Table S1). The problem formulation is given in Equation 1. Where 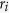 are reactions 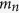 are metabolites forming the stoichiometric matrix *S*. A flux vector **v** is predicted that fulfills the constraints given by the upper, *ub*, and lower bounds, *lb*. An optimization using a vector of objectives, **c**, that normally contains only zeros, apart from for the biomass or substrate reactions.


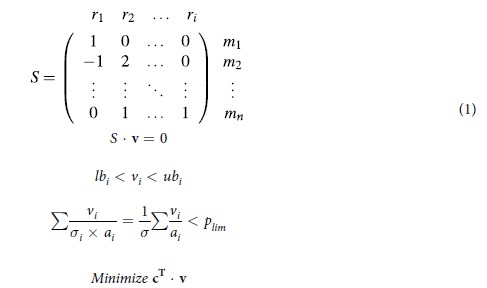


For the visualization of the model (Fig. 1), the co factor metabolites ATP, ADP, NAD, NADH, NADP, NADPH, acetyl-CoA, -CoA, were removed from the stoichiometric matrix *S* and an adjacency matrix was constructed by considering all reactions sharing a metabolite as adjacent. The network was visualized using Cytoscape^58^ and the specific activities of the enzymes were mapped to intensities using the normalized, log transformed, specific activity.

### Analysis of Proteomics

The reference enzyme mass distribution was calculated from proteomics data from literature. The proteomics data^18^ was measured in the mid exponential phase of cells grown on yeast nitrogen base (YNB) liquid medium. The cells were auxotroph for lysine and arginine and the medium supplemented with lysine and arginine. The relative protein abundances were taken from the protein abundance database PaxDb^59^, these abundances were multiplied with tabulated enzyme masses from UniProt^33^. The mass of protein covered by the model, including isozymes, relative to the total protein pool, was calculated as described above. This resulted in an estimated 20% of the protein pool being covered by the model. Together with a typical protein content of *S. cerevisiae* of approximately 50%^2^, the mass of protein covered by the model was estimated to 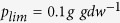. The protein mass covered by protein, amino acid and nucleotide synthesis was calculated using the same method. The respective cellular functions were identified using KEGG^57^ and Uniprot^33^.

### Simulations

The metabolic requirements for ATP production (Fig. 2a), were predicted by incrementally increasing the ATP production and minimizing glucose uptake for each chosen rate. Similarly, for growth (Fig. 2b), the specific growth rate was incrementally increased. To avoid an over prediction of acetate the upper bound on excretion was set to the biologically observed^2^ maximum level, 0.6 mMol/gdw/h (Supplementary Figure 3). In addition, the growth related ATP maintenance drain was varied with the growth rate to fit the experimentally^2^ observed levels (Supplementary Figure S3). The level was increased linearly from 21 to 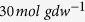 between the growth rates 0.01 and 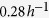 and then linearly decreased to 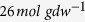 until 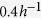
(Supplementary Figure S3). The experimental levels were calculated from exchange fluxes as a sum of contributions from oxygen uptake and fermentation products, 2.75 mole ATP per mole *O*_2_ uptake and 1 mole ATP per mole fermentation product. The fitting of the ATP expenditure was inspired by the shifts in cellular protein content that followed a similar trend.

For the prediction of carbon source dependent maximum growth rates (Fig. 5), chemostat experiments were performed *in silico* with different carbon sources as described above. The protein efficiency was calculated by dividing the estimated protein with the growth rate, at each point.

For the simulation of growth in batch (Supplementary table S5), the growth rate was maximized, with unlimited glucose uptake rate. Simulations where run with and without the addition of an uncoupling reaction, allowing free movement of protons from the inter membrane space to the matrix. The acetate and glycerol excretion was fixed to the experimentally determined levels and the growth related ATP was set to 35 mMol/gdw/h while the protein content was set to 40% in the biomass equation. The fluxes from the batch experiment were used for the protein abundance prediction (Fig. 4).

### Calculation of Flux Control Coefficients

The Flux Control Coefficients 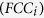, are similar to the ones calculated using Metabolic Control Analysis (MCA). The difference being that the protein abundance determines activity in MCA, and here activity is determined directly by the specific activity parameter. Each specific activity value, 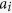, was perturbed by a factor of 1.001 (Eq. 2). The change in predicted growth rate was calculated by comparing the maximum growth rate in the perturbed state 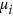 to the non perturbed state *μ* (Eq. 2). The maximum possible value for a 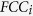 is 1, reflecting a situation where a single enzyme exerts all flux control over the growth rate. To calculate the critical growth rate, the production of ethanol and acetate was constrained to 0. Two objectives were investigated, Biomass production and ATP production. In the case of biomass production on glucose the simulation was run with and without uncoupling.


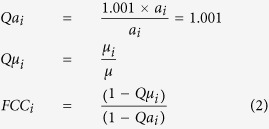


### Estimation of *in vivo* specific activity of F1F0-ATP synthase

For each study (Supplementary Table S4) the *PMF* was calculated from the reported 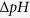 and 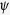 values. The experimental values that were close to 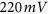 where recorded together with the maximum value found in the study. The experimental turnover values, *s*^−1^, were converted to specific activity, using the molecular mass of F1F0-ATP synthase in yeast.

### Sensitivity analysis and Robustness test

The robustness of the model was investigated by randomly perturbing the specific activity parameters to a degree controlled by a perturbation factor, *I*. For each simulation each specific activity value, 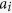, was perturbed by a random value 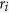 as follows 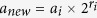, where *r* was sampled from a uniform distribution on the interval −*I* to *I*.

The robustness was further investigated by setting the specific activity to infinity for each enzyme in turn and determining whether ethanol production was still predicted.

## Additional Information

**How to cite this article**: Nilsson, A. and Nielsen, J. Metabolic Trade-offs in Yeast are caused by F1F0-ATP synthase. *Sci. Rep.* **6**, 22264; doi: 10.1038/srep22264 (2016).

## Supplementary Material

Supplementary Information
