# Supplementary material for: Metabolic Trade-offs in Yeast are Caused by F1F0-ATP synthase
Source: Sci Rep. 2016 Mar 1;6:22264. doi: 10.1038/srep22264 (PMC4772093; doi:10.1038/srep22264)
Supplement: Supplementary Information [file srep22264-s1.pdf]

Metabolic Trade-offs in Yeast are caused by F1F0-ATP synthase

Avlant Nilsson and Jens Nielsen

Supplementary Figure S1

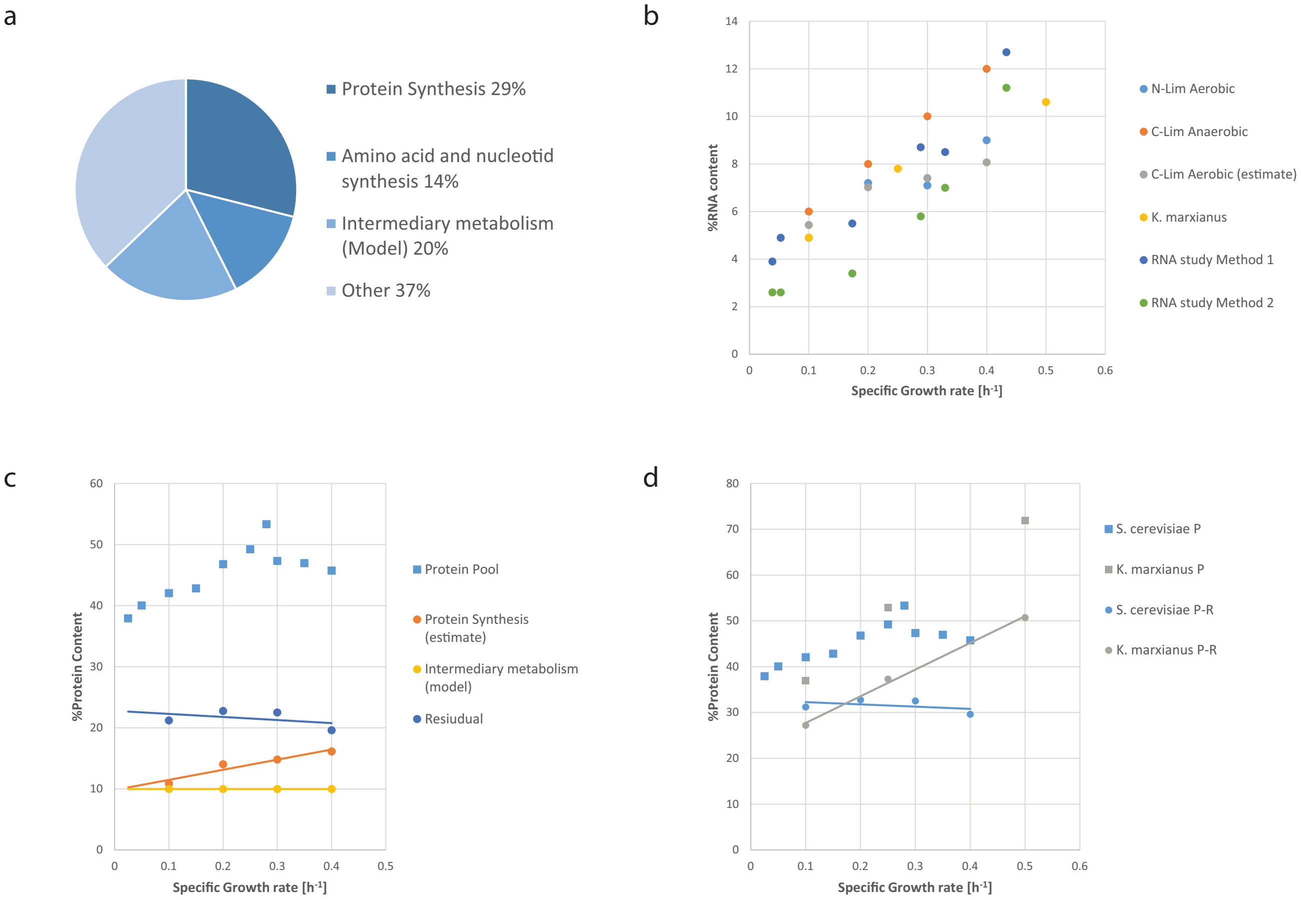

a, The distribution of protein mass between cellular functions, from proteomics data[1, 2] and tabulated enzyme masses[3]. **b** The RNA content as a function of the specific growth rate under; aerobic nitrogen limited conditions in chemostat [4]; anaerobic carbon limited conditions [5] in chemostat; an estimate of the RNA content for under aerobic carbon limited conditions in chemostat, from the protein content [6] and the RNA/Protein ratio at growth rates 0.1, 0.2, 0.3 and 0.4 from the previous conditions; aerobic carbon limited conditions in chemostat for *K. marxianus* [7], and by using substrates with different growth rates, using two different RNA messurment methods [8] . **c,** The growth rate dependent protein content under aerobic glucose limited conditions [6], the share of protein assumed to belong to intermediary metabolism and the fraction of mass in protein syntheses, estimated as $2\times$estimated RNA content. **d,** Comparison of the non-protein synthesizing fraction (Protein - $2\times$RNA content) for *S. cerevisiae* [6] and *K. marxianus* [7].

Supplementary Figure S2

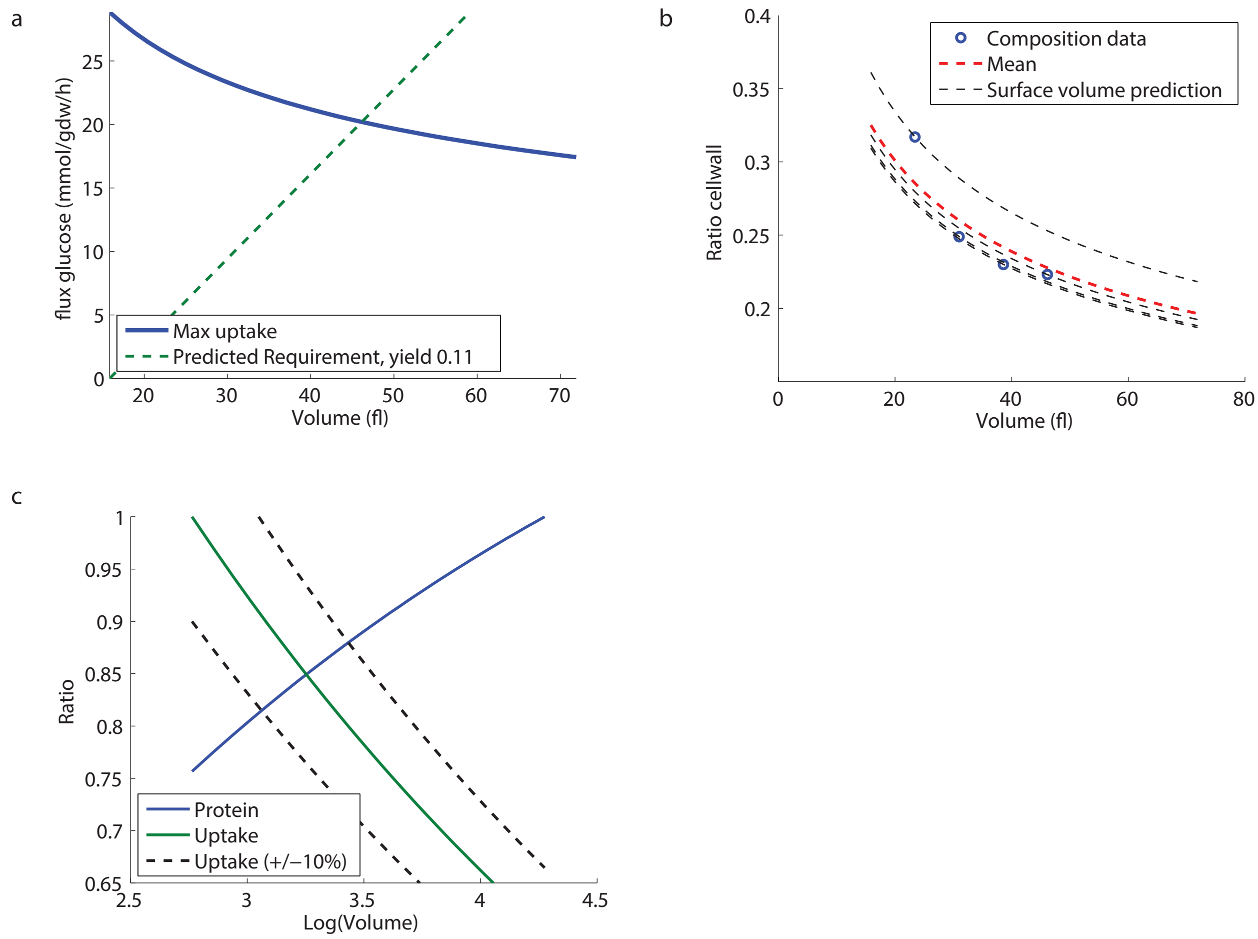

The effects of a decreasing surface/volume ratio when the volume increases for the maximum uptake rate (**a**), the ratio of biomass in the cell wall (**b**), and its implications for the protein content, composition data from [4] (**c**). The maximum uptake rate is assumed to be proportional to the surface/volume ratio and is calculated from an observed uptake rate of 20 mMol/gdw/h at a growth rate of 0.4. The predicted requirement are calculated from a yield of 0.11 gdw/g substrate. The volume is converted to growth rate using an linear regression of volume and growth rate from literature data [9], resulting in the empirical equation $volume = 15.8 + 76.1 \times growthrate$.

Supplementary Table S1

Maximum specific activities for the enzymes in the model were collect from literature. The molecular weight were retried from Uniprot [3]. The reported specific activity was used when available and otherwise calculated from Kcat using the molecular weight. The calculated value is indicated with italics.

Name	EC	Specific Activity $\mu mol\, mg\, min^{-1}$	Kcat s^{-1}	Weight kDa	Source
**Glycolysis**					
HXK	2.7.1.1	310	*274*	53	[10]
PGI	5.3.1.9	675	*685*	61	[11]
PFK	2.7.1.11	180	*642*	214	[12]
FBP	3.1.3.11	110	*69.7*	38	[13]
FBA	4.1.2.13	50	*32.5*	39	[14]
TPI	5.3.1.1	19250	8662.5	27	[15]
GLD	1.2.1.12	40	*24.7*	37	[16]
PGK	2.7.2.3	700	536.7	46	[17]
GPM	5.4.2.11	1280	576	27	[18]
ENO	4.2.1.11	*88.8*	71	48	[19]
CDC	2.7.1.40	*253*	232	55	[20]
**Pentose Phosphate Pathway**					
ZWF	1.1.1.49	678	*632.8*	56	[11]
PGL	3.1.1.31	*22.2*	10	27	[21]
GND	1.1.1.44	42	*36.4*	54	[11]
RPI	5.3.1.6	24	9.6	28	[11]
RPE	5.1.3.1	262	*113.5*	26	[11]
TKLa	2.2.1.1	*36.8*	46	75	[22]
TAL1	2.2.1.2	61	*35.6*	35	[11]
TKLb	2.2.1.1	*55.2*	69	75	[22]
**Fermentation Pathways**					
DAR	1.1.1.8	158	113.0	42.9	[23]
GPP	3.1.3.21	24	11.16	27.9	[24]
PDC	4.1.1.1	60	*62*	62	[25]
ADH1	1.1.1.1	*1421*	900	38	[26]
ALD6	1.2.1.4	24	*22*	55	[27]
**TCA Cycle**					
PYC	6.4.1.1	*27.7*	60	130	[28]
CIT	2.3.3.1	160	*141.3*	53	[11]
ACO	4.2.1.3	100	*143.3*	86	[29]
IDH	1.1.1.41	35.6	*23.7*	40	[11]
IDPH	1.1.1.42	35.6	28	47	[11]
KGD1KGD2	1.2.4.2	7.7	*27.8*	217	[30]
SDH12**	1.3.5.1	151.2	219.3	87	[31]

FRDS2	1.3.1.6	17.8	*15.4*	52	[32]
MDH1	1.1.1.37	308.7	*190.4*	37	[33]
FUM1	4.2.1.2	1150	*1035*	54	[34]
**Oxidative Phosphorylation**					
NDI1	1.6.5.3 (1.6.5.9)	*535.7*	500	56	[35]
NDE2	1.6.5.3 (1.6.5.9)	*500*	500	60	[35]
SDH34**	1.3.5.1	*313.3*	219.3	42	[31]
RIP1	1.10.2.2	*26.7*	220	494	[36]
COX1	1.9.3.1	190	*693.5*	219	[37]
ATP1	3.6.3.14	*11.2*	120	644	[38]
**Galactose**					
GAL1	2.7.1.6	*23.1*	22.3	58	[39]
GAL10	5.1.3.2	31.8	*40.8*	77	[40]
GAL7b	2.7.7.12	54.1	*36.0*	40	[41]
PGM1_2	5.4.2.2	205	*215*	63	[42]
**Other**					
PDH	1.2.4.1	28.9	*83.3*	173	[43]
ACS	6.2.1.1	48	*62.4*	78	[44]
PCK	4.1.1.49	62	62	60	[45]
ALD2	1.2.1.3	34	31.7	56	[46]
ICL1	4.1.3.1	13	13.2	61	[47]*
MLS1	2.3.3.9	18.9	19.2	61	[48]
**Median**		61	71	55	

*Value from expression of ICL1 from Candida tropicali (65% blastp identity) in *S cerevisiae*.

**Kcat for this complex was calculated to 219.3 from a specific activity of 102 and a molecular weight of 129 kDa (67 + 28 + 2x17).

Supplementary Table S2

The fluxes and specific activities for respiration and fermentation were used to estimate the ATP production per gram and minute, assuming that enzymes operate at half maximum specific activity. The sum of flux and flux per ATP is given for comparison.

Reaction	Specific activity*	Flux Respiration	Fermentation	Predicted Mass Respiration	Fermentation
	$mMol\, g^{-1} min^{-1}$	$mMol\, min^{-1}$	$mMol\, min^{-1}$	mg	mg
HXK	310	1	1	6.5	6.5
PGI	675	1	1	3.0	3.0
PFK	180	1	1	11.1	11.1
FBA	50	1	1	40.0	40.0
TPI	19250	1	1	0.1	0.1
GLD	40	2	2	100.0	100.0
PGK	700	2	2	5.7	5.7
GPM	1280	2	2	3.1	3.1
ENO	88.8	2	2	45.1	45.1
CDC	253	2	2	15.8	15.8
PDC	60		2		66.7
ADH1	1421		2		2.8
PDH	27.7	2		144.4	
CIT	160	2		25.0	
ACO	100	2		40.0	
IDH	35.6	2		112.4	
KGD1KGD2	7.7	2		519.5	
SDH12	151.2	2		26.5	
FUM1	1150	2		3.5	
MDH1	308.7	2		13.0	
NDI1	535.7	10		37.3	
SDH34	313.3	2		12.8	
RIP1	26.7	12		898.9	
COX1	190	24		252.6	
ATP1	11.2	13		2321.4	
ATP produced		16.5	2		
Total Mass				4637.5	299.8
(Sum of flux)		(92)	(19)		
(Flux per ATP)		(5.6)	(9.5)		
Specific ATP production $mMol\, g^{-1} min^{-1}$				3.56	6.67
Relative ATP Production				53.4%	187.4%

*See Supplementary Table S1 for references to the specific activity values.

Supplementary Figure S3

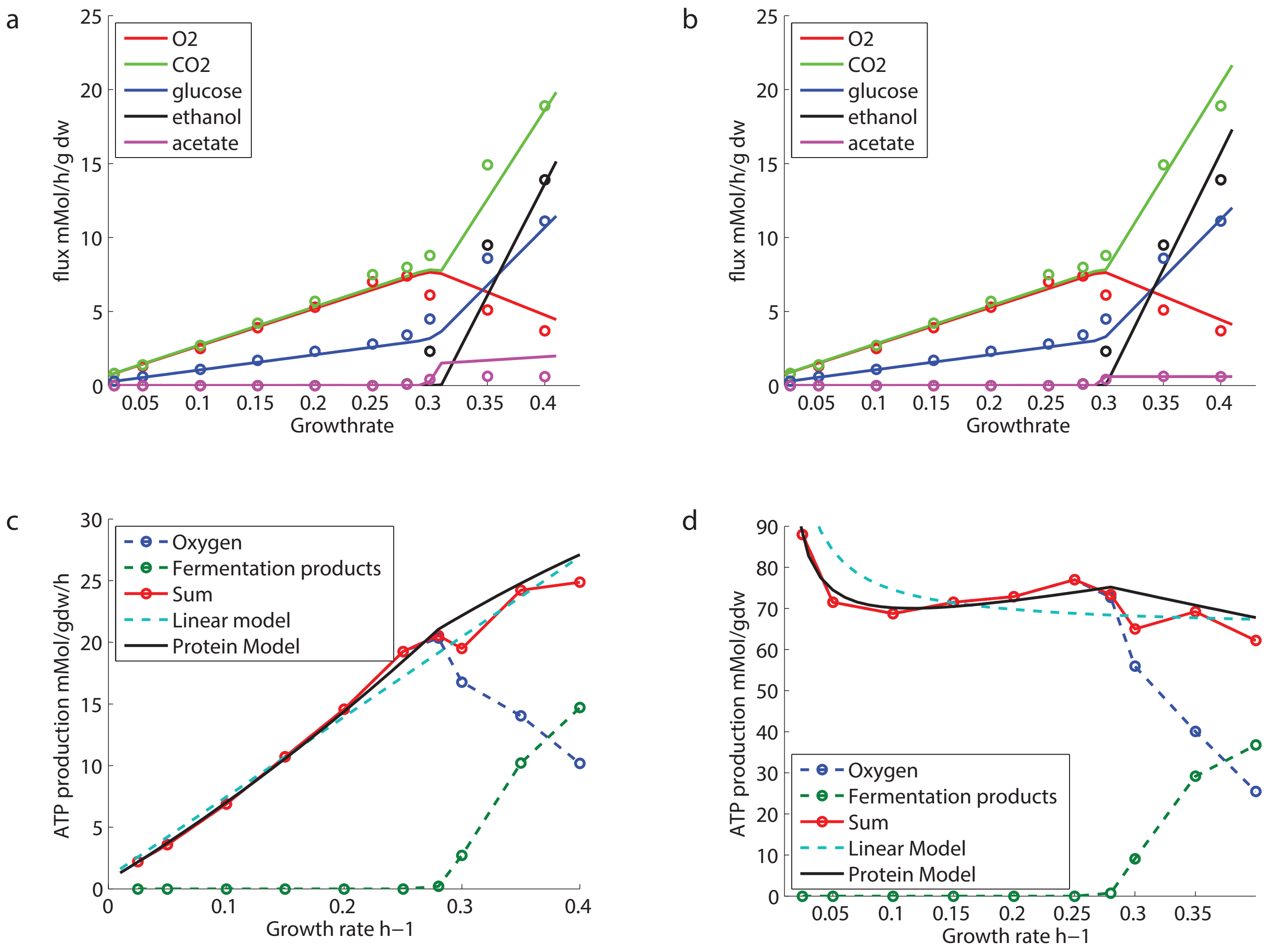

The model fitting process. **a**, original unadjusted predictions, **b**, adjustment of acetate production by imposing a upper bound. The production of ATP calculated from experimental data, compared with a linear regression model and a model where the ATP expenditure is proportional to the protein content represented as fluxes (**c**) and yields (**d**). The linear model was generated by a linear regression of the calculated ATP expenditure and the growth rate, slope $65\,mMol/gdw$ and intersect $1\,mMol/gdw/h$. Experimental values [6] are given as circles and model predictions as lines.

Supplementary Table S3

Flux Control Coefficients for maximization of biomass and for production of ATP on different carbon sources.

**Reaction**	**Critical dilution rate**		**Glucose**			**Galactose**		**Ethanol**		**Acetate**	
	Biomass	ATP	Biomass	ATP	BWoD*	Biomass	ATP	Biomass	ATP	Biomass	ATP
GAL1						0.133	0.182				
GAL10						0.096	0.132				
GAL7b						0.057	0.078				
PGM1_2						0.015	0.021				
HXK	0.003	0.001	0.02	0.022	0.016						
PGI	0.001	0.001	0.008	0.01	0.006	0.004	0.006				
PFK	0.004	0.003	0.031	0.038	0.024	0.015	0.023				
FBP								0.001		0.001	
FBA	0.014	0.009	0.11	0.136	0.086	0.056	0.084				
TPI	0	0	0	0	0	0	0				
GLD	0.035	0.023	0.277	0.339	0.216	0.138	0.21				
PGK	0.002	0.001	0.016	0.019	0.012	0.008	0.012				
GPM	0.001	0.001	0.009	0.011	0.007	0.004	0.007				
ENO	0.016	0.01	0.124	0.153	0.097	0.062	0.095				
CDC	0.005	0.004	0.043	0.054	0.033	0.021	0.033	0		0	
ZWF	0		0.001		0.001	0		0		0	
PGL	0.011		0.019		0.017	0		0		0.004	
GND	0.006		0.01		0.009					0.002	
RPI	0.005		0.007		0.007	0.001		0.001		0.002	
RPE	0.001		0.001		0.001					0	
TKLa	0.002		0.004		0.003	0		0		0.001	
TAL1	0.001		0.002		0.002	0		0		0	
TKLb	0.001		0.002		0.001					0	
PDC	0.001		0.165	0.226	0.127	0.08	0.14				
ADH			0.007	0.01	0.005	0.003	0.006				
ALD6	0.002				0.003	0.023		0.011			
ALD2								0.059	0.092		
ICL	0.001							0.04		0.029	
MLS1	0.001							0.027		0.02	
PDH	0.031	0.031	0.016		0.013	0.009					
ACS	0.001				0.002	0.001		0.025		0.039	0.028
PCK								0.007		0.005	
PYC	0.006		0.01		0.009	0.007					
CIT	0.005	0.006	0.001		0.001	0.001		0.004		0.009	0.008
ACO	0.007	0.009	0.001		0.001	0.001		0.006		0.014	0.013
IDH	0.018	0.026								0.029	0.038
IDPH	0.003		0.004		0.004	0.003		0.001		0.001	

KGD	0.091	0.118	0.016		0.015	0.01		0.005		0.139	0.175
SDH12	0.005	0.006	0.001		0.001	0.001		0.004		0.01	0.009
FUM1	0.001	0.001	0		0	0		0		0.001	0.001
MDH1	0.002	0.003	0		0	0		0.003		0.006	0.004
NDI1	0.008	0.008	0.004		0.004	0.003		0.01	0.012	0.007	0.008
SDH34	0.002	0.003	0		0	0		0.002		0.005	0.004
RIP	0.197	0.204	0.084		0.078	0.073		0.214	0.235	0.195	0.202
COX	0.055	0.057	0.024		0.022	0.02		0.06	0.066	0.055	0.057
ATP	0.488	0.508			0.194	0.184		0.572	0.632	0.497	0.512

*Biomass without uncoupling

Supplementary Table S4

Catalytic activity, k_{cat}, of ATP synthase, under biological and optimal conditions. The biologically conditions refer to a temperature of around 30 C and a protein motive force of around 220 mV [49]. The optimal conditions refers to the maximum value obtained in the study.

Organism and method	Biological rate s^{-1}	Max rate s^{-1}	Reference
E. coli	50	270	Etzold et al. [50]
E. coli	66	66	Iino et al. [51]
E.coli	5	65	Kaim and Dimroth [52]
P.modestum	40	40	Kaim and Dimroth [52]
Spinach (Cloroplasts)	80	160	Kaim and Dimroth [52]
Rhodospirillum rubrum	80	200	Slooten and Vandenbranden [53]
Not specified (Cloroplasts)	66*	200	Schmidt and Gräber [54]
Bacillus PS3	23	23	Soga et al. [55]
Bos taurus (heart)	30	440	Matsuno-Yagi and Hatefi [56]
S. cerevisiae	30	120	Förster et al. [38]
Mean	47 ± 26	158 ± 127	

* Estimated from a reported 3 times lower ATP yield for biological values of the proton motive force.

Supplementary Table S5

Fluxes from chemostat [6] and batch culture [57] are compared with a simulated batch experiment with and without uncoupling. The ATP production rate was calculated as $X \times O_2 + ethanol + acetate + glycerol$, where X was 0.95 for the uncoupled state and 2.75 for the coupled.

	Unit	Experiment		Batch Model	
		Batch	Chemostat	W uncoupling	W/O uncoupling
o2	$mmol\, gdw^{-1} h^{-1}$	2.9*	3.7	3.1	2.8
co2	$mmol\, gdw^{-1} h^{-1}$	33.8	18.9	31.6	25.3
glucose	$mmol\, gdw^{-1} h^{-1}$	19.9	11.1	18.3	15.1
ethanol	$mmol\, gdw^{-1} h^{-1}$	29.6	13.9	27.6	21.5
acetate	$mmol\, gdw^{-1} h^{-1}$	1.3	0.6	1.3**	1.3**
glycerol	$mmol\, gdw^{-1} h^{-1}$	1.7	0.15	1.7**	1.7**
Growth Rate	h^{-1}	0.4	0.4	0.4	0.38
Yield	$\frac{gdw}{g\, glucose}$	0.11	0.20	0.12	0.14
ATP production	$mmol\, gdw^{-1} h^{-1}$	40.6	24.8		32.2
ATP production (uncoupled)	$mmol\, gdw^{-1} h^{-1}$	35.4		33.5	

* Calculated from co2-ethanol-acetate

** These values were constrained to fit experimental values

Supplementary Table S6

The mass ratio calculated from protein abundance data compared with the model predictions. The model was simulating the same batch experiment as in Supplementary Table S5.

Reaction	%Mass Proteomics	%Mass Model
PDC	14.387	16.059
GLD	10.119	26.767
ENO	9.279	12.013
PGK	8.833	1.523
FBA	7.696	11.297
ADH1	6.275	0.648
PFK	4.573	3.138
ACO	4.384	0.123
GPM	4.010	0.833
CDC	3.959	4.163
PGI	3.913	0.826
ALD6	3.501	1.806
HXK	2.648	1.971
TKL	2.457	0.000
PYC	1.634	0.902
GND	1.508	0.426
ATP1	1.443	0.000
GPP	1.440	2.361
ALD2	1.113	0.000
PDH	0.993	1.542
TPI	0.977	0.026
TAL1	0.746	0.088
ACS	0.736	0.000
IDH+IDPH	0.697	0.344
DAR	0.367	0.359
KGD1KGD2	0.362	1.372
RIP1	0.354	7.402
FRDS2	0.336	0.000
CIT	0.263	0.077
ZWF	0.254	0.028
FUM1	0.190	0.009
MDH1	0.166	0.034
NDI1	0.103	0.349
SDH12	0.100	0.070
PGL	0.080	0.858
RPE	0.036	0.028
RPI	0.031	0.444

COX1	0.027	2.080
SDH34	0.005	0.034
MLS1	0.001	0.000
FBP	0.000	0.000
ICL1	0.000	0.000
PCK	0.000	0.000
NDE	0.000	0.000

Supplementary Figure S4

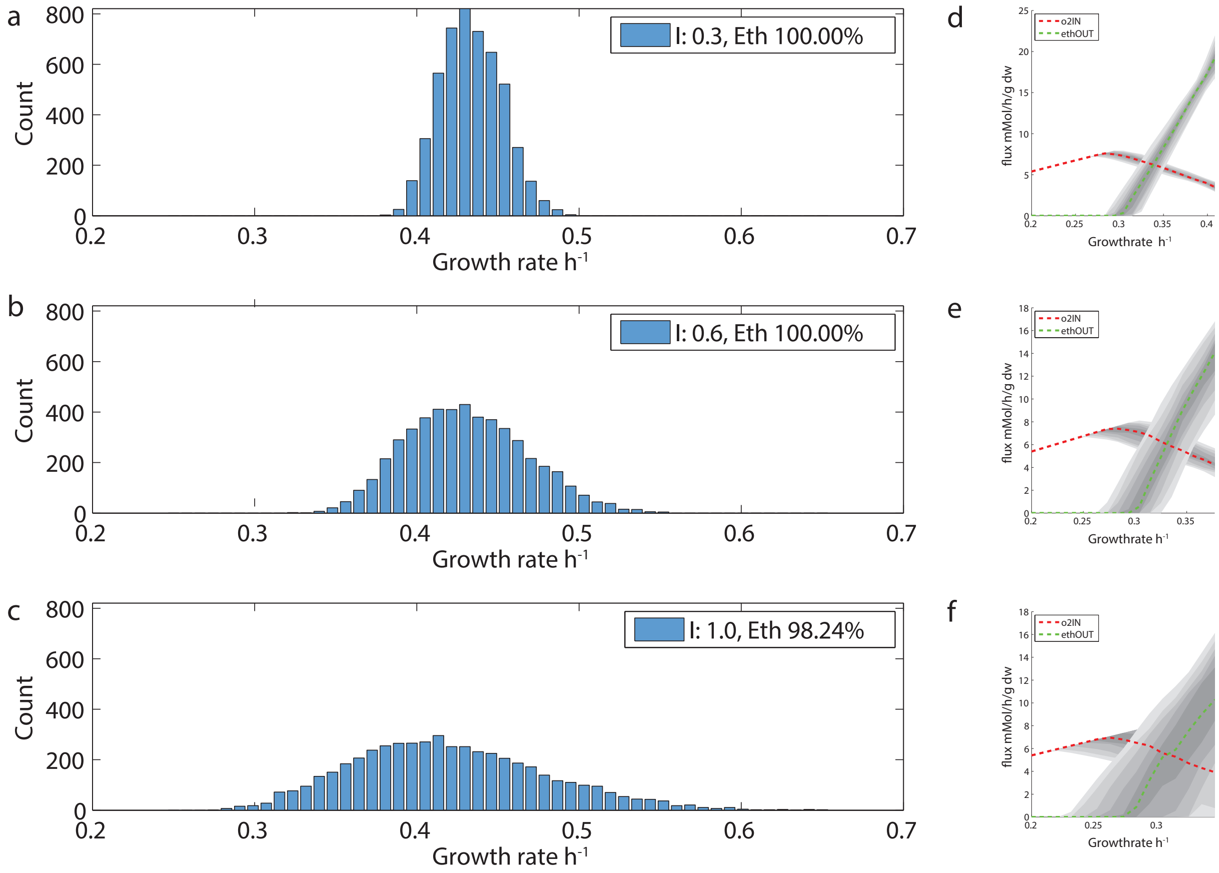

Histograms (**a**, **b** and **c**) of the predicted growth rate when randomly perturbing the specific activity values with an increasing perturbation factor, I, where the value 0.3 corresponds to a perturbation with at most 23%, 0.6 to 52% and 1 to 100%. The distributions are the result of 5 000 simulations with such perturbations. The percentage of simulations that predict ethanol (Eth) production is given in the legend. The median and percentiles (**d**, **e**, and **f**) of the oxygen and ethanol flux from 100 simulations. Percentiles between 25 and 75 are displayed in gray with 5 percentile increment.

SI References

References

[1] Wang M *et al.* PaxDb, a Database of Protein Abundance Averages Across All Three Domains of Life. *Molecular & Cellular Proteomics*, **11**:492–500, 2012.

[2] de Godoy L. M. F *et al.* Comprehensive mass-spectrometry-based proteome quantification of haploid versus diploid yeast. *Nature*, **455**(7217):1251–1254, oct 2008.

[3] UNIPROT. Activities at the Universal Protein Resource (UniProt). *Nucleic acids research*, **42**:D191–8, 2014.

[4] Schulze U. *Anaerobic physiology of Saccharomyces cerevisiae*. PhD thesis, Lyngby, Technical University of Denmark, 1995.

[5] Nissen T. L, Schulze U, Nielsen J, Villadsen J. Flux distributions in anaerobic, glucose-limited continuous cultures of Saccharomyces cerevisiae. *Microbiology*, **143 (Pt 1**:203–218, 1997.

[6] van Hoek P, van Dijken J. P, Pronk J. T. Effect of specific growth rate on fermentative capacity of baker's yeast. *Appl.Environ.Microbiol.*, **64**:4226–4233, 1998.

[7] Fonseca G. G, Gombert A. K, Heinzle E, Wittmann C. Physiology of the yeast Kluyveromyces marxianus during batch and chemostat cultures with glucose as the sole carbon source. *FEMS Yeast Research*, **7**:422–435, 2007.

[8] Waldron C, Lacroute F. Effect of growth rate on the amounts of ribosomal and transfer ribonucleic acids in yeast. *Journal of bacteriology*, **122**:855–865, 1975.

[9] Boer V. M, Crutchfield C. A, Bradley P. H, Botstein D, Rabinowitz J. D. Growth-limiting intracellular metabolites in yeast growing under diverse nutrient limitations. *Molecular biology of the cell*, **21**:198–211, 2010.

[10] Jacob L, Beecken V, Bartunik L. J, Rose M, Bartunik H. D. Purification and crystallization of yeast hexokinase isoenzymes: Characterization of different forms by chromatofocusing. *Journal of Chromatography A*, **587**(1):85–92, 1991.

[11] Albe K. R, Butler M. H, Wright B. E. Cellular concentrations of enzymes and their substrates. *Journal of theoretical biology*, **143**:163–195, 1990.

[12] Welch P, Scopes R. K. Rapid purification and crystallization of yeast phosphofructokinase. *Analytical Biochemistry*, **112**(1): 154–157, 1981.

[13] Marcus F *et al.* Yeast (Saccharomyces cerevisiae) fructose-1,6-bisphosphatase. Properties of phospho and dephospho forms and of two mutants in which serine 11 has been changed by site-directed mutagenesis. *Journal of Biological Chemistry*, **263** (13):6058–6062, 1988.

[14] White J. S, White D. C. *Source Book of Enzymes*. CRC Press, 1997.

[15] Nickbarg E. B, Knowles J. R. Triosephosphate isomerase: energetics of the reaction catalyzed by the yeast enzyme expressed in Escherichia coli. *Biochemistry*, **27**(16):5939–5947, 1988.

[16] BROWNLEE A. G, PHILLIPS D. R, POLYA G. M. Purification and Characterization of Two High-Affinity (Adenosine 3',5'-Monophosphate)-Binding Proteins from Yeast. *European Journal of Biochemistry*, **109**(1):39–49, 1980.

[17] McHarg J, Kelly S. M, Price N. C, Cooper A, Littlechild J. A. Site-directed mutagenesis of proline 204 in the 'hinge' region of yeast phosphoglycerate kinase. *European Journal of Biochemistry*, **259**(3):939–946, 1999.

[18] SASAKI R, UTSUMI S, SUGIMOTO E, CHIBA H. Subunit Structure and Multifunctional Properties of Yeast Phosphoglyceromutase. *European Journal of Biochemistry*, **66**(3):523–533, 1976.

[19] Kornblatt M. J *et al.* The Saccharomyces cerevisiae enolase-related regions encode proteins that are active enolases. *Yeast*, **30**(2):55–69, 2013.

[20] Susan-Resiga D, Nowak T. Proton Donor in Yeast Pyruvate Kinase: Chemical and Kinetic Properties of the Active Site Thr 298 to Cys Mutant. *Biochemistry*, **43**(48):15230–15245, 2004.

[21] Messiha H. L *et al.* Enzyme characterisation and kinetic modelling of the pentose phosphate pathway in yeast. *PeerJ PrePrints*, **2**:e146v4, 2014.

[22] Nilsson U, Hecquet L, Gefflaut T, Guerard C, Schneider G. Asp477 is a determinant of the enantioselectivity in yeast transketolase. *FEBS Letters*, **424**(1-2):49–52, 1998.

[23] Merkel J. R, Straume M, Sajer S. A, Hopfer R. L. Purification and some properties of sn-glycerol-3-phosphate dehydrogenase from Saccharomyces cerevisiae. *Analytical Biochemistry*, **122**(1):180–185, 1982.

[24] Norbeck J, Påhlman A.-k, Akhtar N, Blomberg A, Adler L. Purification and Characterization of Two Isoenzymes of DL -Glycerol-3-phosphatase from Saccharomyces cerevisiae. *The Journal of Biological Chemistry*, **271**(23):13875–13881, 1996.

[25] Li H, Furey W, Jordan F. Role of Glutamate 91 in Information Transfer during Substrate Activation of Yeast Pyruvate Decarboxylase. *Biochemistry*, **38**(31):9992–10003, 1999.

[26] Gould R. M, Plapp B. V. Substitution of arginine for histidine-47 in the coenzyme binding site of yeast alcohol dehydrogenase I. *Biochemistry*, **29**(23):5463–5468, 1990.

[27] Dickinson F. M. {T}he purification and some properties of the {M}g(2+)-activated cytosolic aldehyde dehydrogenase of {S}accharomyces cerevisiae. *Biochem. J.*, **315 (Pt 2**:393–399, apr 1996.

[28] Branson J. P, Nezic M, Jitrapakdee S, Wallace J. C, Attwood P. V. Kinetic Characterization of Yeast Pyruvate Carboxylase Isozyme Pyc1 and the Pyc1 Mutant, C249A. *Biochemistry*, **43**(4):1075–1081, 2004.

[29] SUZUKI T *et al.* Crystallization and Reconstitution of Yeast Aconitase. *Agricultural and Biological Chemistry*, **37**(9): 2211–2212, 1973.

[30] Hirabayashi T, Harada T. Isolation and properties of α-ketoglutarate dehydrogenase complex from baker's yeast (Saccharomyces cerevisiae). *Biochemical and Biophysical Research Communications*, **45**(6):1369–1375, 1971.

[31] Oyedotun K. S, Lemire B. D. The Quinone-binding Sites of the Saccharomyces cerevisiae Succinate-ubiquinone Oxidoreductase. *Journal of Biological Chemistry*, **276**(20):16936–16943, 2001.

[32] Muratsubaki H, Enomoto K. {O}ne of the fumarate reductase isoenzymes from {S}accharomyces cerevisiae is encoded by the {O}{S}{M}1 gene. *Arch. Biochem. Biophys.*, **352**(2):175–181, apr 1998.

[33] McAlister-Henn L, Thompson L. M. Isolation and expression of the gene encoding yeast mitochondrial malate dehydrogenase. *Journal of Bacteriology*, **169**(11):5157–5166, 1987.

[34] Keruchenko J. S, Keruchenko I. D, Gladilin K. L, Zaitsev V. N, Chirgadze N. Y. Purification, characterization and preliminary X-ray study of fumarase from Saccharomyces cerevisiae. *Biochimica et Biophysica Acta (BBA) - Protein Structure and Molecular Enzymology*, **1122**(1):85–92, 1992.

[35] de VRIES S, GRIVELL L. A. Purification and characterization of a rotenone-insensitive NADH: Q6 oxidoreductase from mitochondria of Saccharomyces cerevisiae. *European Journal of Biochemistry*, **176**(2):377–384, 1988.

[36] Kessl J. J *et al.* Cytochrome b Mutations That Modify the Ubiquinol-binding Pocket of the Cytochrome bc1 Complex and Confer Anti-malarial Drug Resistance in Saccharomyces cerevisiae. *Journal of Biological Chemistry*, **280**(17):17142–17148, 2005.

[37] Mason T. L, Poyton R. O, Wharton D. C, Schatz G. Cytochrome c Oxidase from Bakers' Yeast : I. ISOLATION AND PROPERTIES. *Journal of Biological Chemistry*, **248**(4):1346–1354, 1973.

[38] Förster K *et al.* Proton transport coupled ATP synthesis by the purified yeast H+-ATP synthase in proteoliposomes. *Biochimica et Biophysica Acta - Bioenergetics*, **1797**:1828–1837, 2010.

[39] Sellick C. A, Reece R. J. Contribution of amino acid side chains to sugar binding specificity in a galactokinase, Gal1p, and a transcriptional inducer, Gal3p. *The Journal of biological chemistry*, **281**:17150–17155, 2006.

[40] Fukasawa T, Obonai K, Segawa T, Nogi Y. The enzymes of the galactose cluster in Saccharomyces cerevisiae. II. Purification and characterization of uridine diphosphoglucose 4-epimerase. *Journal of Biological Chemistry*, **255**(7):2705–2707, 1980.

[41] Segawa T, Fukasawa T. The enzymes of the galactose cluster in Saccharomyces cerevisiae. Purification and characterization of galactose-1-phosphate uridylyltransferase. *Journal of Biological Chemistry*, **254**(21):10707–10709, 1979.

[42] DAUGHERTY J. P, KRAEMER W. F, JOSHI J. G. Purification and Properties of Phosphoglucomutase from Fleischmann's Yeast. *European Journal of Biochemistry*, **57**(1):115–126, 1975.

[43] KRESZE G.-B, RONFT H. Pyruvate Dehydrogenase Complex from Baker's Yeast. *European Journal of Biochemistry*, **119** (3):573–579, 1981.

[44] Satyanarayana T, Klein H. P. Studies on the "aerobic" acetyl-coenzyme a synthetase of Saccharomyces cerevisiae: Purification, crystallization, and physical properties of the enzyme. *Archives of Biochemistry and Biophysics*, **174**(2):480–490, 1976.

[45] Castillo D, Sepúlveda C, Cardemil E, Jabalquinto A. M. Functional evaluation of serine 252 of Saccharomyces cerevisiae phosphoenolpyruvate carboxykinase. *Biochimie*, **91**(2):295–299, 2009.

[46] Bostian K. A, Betts G. F. {R}apid purification and properties of potassium-activated aldehyde dehydrogenase from {S}accharomyces cerevisiae. *Biochem. J.*, **173**(3):773–786, sep 1978.

[47] Oda K *et al.* {H}igh level expression of isocitrate lyase gene of n-alkane-utilizing yeast {C}andida tropicalis in {S}accharomyces cerevisiae. *Arch. Microbiol.*, **156**(6):439–443, 1991.

[48] Okada H, Ueda M, Tanaka A. Purification of peroxisomal malate synthase from alkane-grown Candida tropicalis and some properties of the purified enzyme. *Archives of Microbiology*, **144**(2):137–141, 1986.

[49] Perry S. W, Norman J. P, Barbieri J, Brown E. B, Gelbard H. A. Mitochondrial membrane potential probes and the proton gradient: A practical usage guide. *BioTechniques*, **50**:98–115, 2011.

[50] Etzold C, Deckers-Hebestreit G, Altendorf K. Turnover number of Escherichia coli F0F1 ATP synthase for ATP synthesis in membrane vesicles. *European journal of biochemistry / FEBS*, **243**:336–343, 1997.

[51] Iino R, Hasegawa R, Tabata K. V, Noji H. Mechanism of inhibition by C-terminal α-helices of the ϵ subunit of Escherichia coli FoF1-ATP synthase. *Journal of Biological Chemistry*, **284**:17457–17464, 2009.

[52] Kaim G, Dimroth P. ATP synthesis by F-type ATP synthase is obligatorily dependent on the transmembrane voltage. *EMBO Journal*, **18**:4118–4127, 1999.

[53] Slooten L, Vandenbranden S. ATP-synthesis by proteoliposomes incorporating Rhodospirillum rubrum F0F1 as measured with firefly luciferase: dependence on delta psi and delta pH. *Biochimica et biophysica acta*, **976**:150–160, 1989.

[54] Schmidt G, Gräber P. The rate of ATP synthesis catalyzed by reconstituted CF0F1-liposomes: Dependence on ΔpH and $\Delta\psi$. *Biochimica et Biophysica Acta (BBA) - Bioenergetics*, **890**:392–394, 1987.

[55] Soga N, Kinosita K, Yoshida M, Suzuki T. Kinetic equivalence of transmembrane ph and electrical potential differences in ATP synthesis. *Journal of Biological Chemistry*, **287**:9633–9639, 2012.

[56] Matsuno-Yagi A, Hatefi Y. Estimation of the turnover number of bovine heart F0F1 complexes for ATP synthesis. *Biochemistry*, **27**:335–340, 1988.

[57] Heyland J, Fu J, Blank L. M. Correlation between TCA cycle flux and glucose uptake rate during respiro-fermentative growth of Saccharomyces cerevisiae. *Microbiology*, **155**:3827–3837, 2009.
